# Bone Marrow Suppression during Postoperative Radiation for Bladder Cancer and Comparative Benefit of Proton Therapy—Phase 2 Trial Secondary Analysis

**DOI:** 10.14338/IJPT-21-00003.1

**Published:** 2021-07-06

**Authors:** Robert H. Press, Joseph W. Shelton, Chao Zhang, Quang Dang, Sibo Tian, Timothy Shu, Crystal S. Seldon, Shaakir Hasan, Ashesh B. Jani, Jun Zhou, Mark W. McDonald

**Affiliations:** 1 New York Proton Center, New York, NY, USA; 2 Department of Radiation Oncology, Winship Cancer Institute of Emory University, Atlanta, GA, USA; 3 Biostatistics Core of Department Pediatrics, Emory University, Atlanta, GA, USA; 4 Department of Radiation Oncology, University of Miami Miller School of Medicine, Sylvester Comprehensive Cancer Center, Miami, FL, USA

**Keywords:** urinary bladder neoplasms, lymphopenia, bone marrow suppression, cystectomy, proton therapy

## Abstract

**Purpose:**

For patients with high-risk bladder cancer (pT3^+^ or N^+^), local regional failure remains a challenge after chemotherapy and cystectomy. An ongoing prospective phase 2 trial (NCT01954173) is examining the role of postoperative photon radiation therapy for high-risk patients using volumetric modulated arc therapy. Proton beam therapy (PBT) may be beneficial in this setting to reduce hematologic toxicity. We evaluated for dosimetric relationships with pelvic bone marrow (PBM) and changes in hematologic counts before and after pelvic radiation therapy and explored the potential of PBT treatment plans to achieve reductions in PBM dose.

**Materials and Methods:**

All enrolled patients were retrospectively analyzed after pelvic radiation per protocol with 50.4 to 55.8 Gy in 28 to 31 fractions. Comparative PBT plans were generated using pencil-beam scanning and a 3-beam multifield optimization technique. Changes in hematologic nadirs were assessed using paired *t* test. Correlation of mean nadirs and relative PBM dose levels were assessed using the Pearson correlation coefficient (CC).

**Results:**

Eighteen patients with a median age of 70 were analyzed. Mean cell count values after radiation therapy decreased compared with preradiation therapy values for white blood cells (WBCs), absolute neutrophil count (ANC), absolute lymphocyte count (all *P* < .001), and platelets (*P* = .03). Increased mean PBM dose was associated with lower nadirs in WBC (Pearson CC −0.593, *P* = .02), ANC (Pearson CC −0.597, *P* = .02), and hemoglobin (Pearson CC −0.506, *P* = .046), whereas the PBM V30 to V40 correlated with lower WBC (Pearson CC −0.512 to −0.618, *P* < .05), and V20 to V30 correlated with lower ANC (Pearson CC −0.569 to −0.598, *P* < .04). Comparative proton therapy plans decreased the mean PBM dose from 26.5 Gy to 16.1 Gy (*P* < .001) and had significant reductions in the volume of PBM receiving doses from 5 to 40 Gy (*P* < .001).

**Conclusion:**

Increased PBM mean dose and V20 to V40 were associated with lower hematologic nadirs. PBT plans reduced PBM dose and may be a valuable strategy to reduce the risk of hematologic toxicity in these patients.

## Introduction

The current standard of care for locally advanced urothelial bladder carcinoma is neoadjuvant chemotherapy, followed by radical cystectomy. However, patients with high-risk disease, defined as stages pT3 and T4 or positive pelvic lymph nodes, continue to have a substantial risk of local regional failure (LRF), despite that treatment paradigm. In SWOG (formerly the Southwest Oncology Group, Portland, Oregon) 8710, a randomized trial of radical cystectomy with or without neoadjuvant MVAC (methotrexate, vinblastine sulfate, doxorubicin hydrochloride [adriamycin], and cisplatin) chemotherapy, the 5-year cumulative incidence of LRF for patients with ≥ pT3^+^ disease was 32% [1]. Other retrospective series, including a Cochrane meta-analysis, report LRF rates ranging from 26% to 44% [2–4].

Pelvic recurrences can be associated with significant morbidity, including urinary and bowel obstructions, hematuria, and pain. Randomized prospective trials investigating the use of chemotherapy have not shown improvements in LRF, and salvage outcomes remain poor. The use of adjuvant radiation therapy has, therefore, been studied to address this pattern of failure [5–7]. Despite historical randomized data supporting improvements in local regional control with the use of adjuvant radiation therapy (RT), this treatment paradigm has not been widely adopted because of concerns of excessive toxicity in an often-frail patient population.

**Figure 1.**
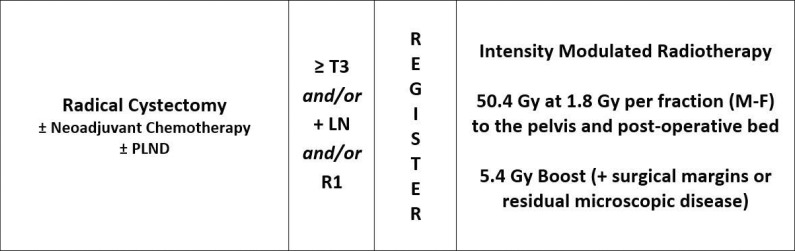
Schema of NCT01954173 phase 2 trial [8].

Modern radiation techniques are now more capable of improving the therapeutic window. An ongoing prospective phase 2 trial (NCT01954173) [8] is examining the efficacy and safety of postoperative photon RT for high-risk patients using volumetric modulated arc therapy (VMAT) (**Figure 1**). In addition to gastrointestinal and urinary toxicity, pelvic RT is also associated with hematologic suppression. Prior studies in other pelvic malignancies have found correlations between radiation dose to pelvic bone marrow (PBM) and acute hematologic toxicity [9,10] In this clinical context, proton beam therapy (PBT) is uniquely capable of treating pelvic lymph nodes and the cystectomy bed while minimizing excess dose to PBM and may, therefore, be beneficial in this disease setting. This study subsequently sought to examine the enrolled patients on the previously mentioned prospective trial [8] for potential dosimetric correlations between the PBM dose and acute myelosuppression and to quantify the potential of PBT treatment plans to achieve reductions in PBM dose compared with the delivered VMAT plans.

## Materials and Methods

This study was approved by our institutional review board. NCT01954173 [8] is a prospective phase 2 single-institution trial enrolling patients with pT3 and T4, node-positive, and/or positive margins after radical cystectomy for bladder cancer. Systemic therapy is allowed either in the neoadjuvant or adjuvant setting, but no chemotherapy is administered concurrently with the radiation therapy. For this analysis, the outcomes of patients enrolled to date were retrospectively analyzed after pelvic radiation of 50.4 or 55.8 Gy in 28 or 31 fractions per protocol. Patients underwent computed tomography simulation in the supine position and immobilized via Vac-Lok cushions (CIVCO, Coralville, Iowa). Protocol-directed clinical target volume (CTV) included obturator, internal and external iliac, common iliac, and presacral lymph nodes, as well as the cystectomy bed for patients with R1 resection. A 7-mm margin was added to the CTV to create the planning target volume (PTV) to account for setup error. Treatment was delivered using VMAT technique for all patients. Image guidance included daily orthogonal kilovolt x-rays and daily cone-beam computed tomographies, matching to bony anatomy. Required organ-at-risk dose constraints included small bowel volume receiving a dose of 40 Gy (V40) < 30%, rectum V45 < 60%, and femoral heads V30 < 15%. Volume-based PBM dose constraints were not required per the protocol.

Complete blood cell counts (CBCs) before and immediately after the completion of radiation therapy were reviewed. Pretreatment laboratory tests were completed within 2 weeks before starting radiation therapy and posttreatment laboratory tests were completed within 2 weeks after radiation therapy completion. Laboratory results were not checked routinely during the radiation therapy course. Nadirs of white blood cell (WBC), absolute neutrophil count (ANC), absolute lymphocyte count (ALC), hemoglobin (Hgb), and platelet counts were recorded. For the basis of this study, PBM was delineated by contouring all pelvic bones, including the bilateral ilium, ischium, sacrum, and pubis, as well as the proximal femur, cropping out the cortical bone with an automated inner margin of 5 mm. Per protocol, the CTV to PTV expansion was 0.7 cm. Comparative pencil-beam scanning PBT plans were generated using a 3-beam multifield-optimization technique with 2 opposed lateral fields and a posteroanterior field. Plans were developed to treat the predefined protocol CTV with robust optimization to include an additional 0.5 cm of setup uncertainties in the *x*, *y*, and *z* directions and a 4% range uncertainty. Beams were generally weighted 2:1:1 in favor of the posteroanterior field. Beam-specific targets were generated so that lateral beams covered only the corresponding ipsilateral targets. Intended CTV coverage was V100% ≥ 98%. A density override to water equivalence was performed on areas of bowel gas. The plans were robustly optimized using the RayStation treatment planning system (version 8A, RaySearch Laboratories, Stockholm, Sweden). Similar to the delivered VMAT plans, no volume-based PBM constraints were used in optimization, instead, prioritizing robust coverage of the target to match the VMAT plans. Dose in grays was prescribed using a relative biological effectiveness of 1.1 times.

Descriptive statistics of patient, disease, and treatment characteristics were reported. For numeric covariates, the mean and SD were calculated. Changes in CBC nadirs for each cell type were assessed using a paired *t* test. Correlation of mean CBC nadirs and relative PBM dose levels were assessed using the Pearson correlation coefficient (CC). A negative Pearson CC indicates a lower cell-count nadir from baseline. Radiation dose levels examined include mean dose, V5, V10, V20, V30, and V40. Comparisons of organ-at-risk dose metrics between photon and PBT plans were performed using absolute volume (cubic centimeters) via the 1-way analysis of variance and Kruskal-Wallis tests. The threshold for significance was set at *P* < .05. Statistical analysis was conducted using SAS version 9.4 software (SAS Institute, Cary, North Carolina).

## Results

From October 2013 to December 2018, 18 patients were enrolled and were eligible for this analysis. The median age was 70 years (range, 47-82 years), and most patients received neoadjuvant chemotherapy (66.1%). Two patients were unable to complete treatment (completed 1 and 3 fractions, respectively) and were excluded from the correlative analysis, but their cases were used for dosimetric comparisons. See Table 1 for complete patient and treatment characteristics.

**Table 1.** Patient and treatment characteristics (N = 18).

**Variable**	**Patients, No. (%)**
Age, mean ± SD, y	68 ± 9
Race	
Black	3 (16.7)
White	15 (83.3)
Sex	
Female	4 (22.2)
Male	14 (77.8)
Histology	
Squamous cell carcinoma	1 (5.6)
Urothelial carcinoma	17 (94.4)
Tumor size, mean ± SD, cm	3.2 ± 2.5
T stage	
pT2	6 (46.2)
pT3a	4 (30.8)
pT3b	1 (7.7)
pT4a	2 (15.4)
Unknown	5
Chemotherapy	
Adjuvant	4 (22.2)
Neoadjuvant	11 (61.1)
Neoadjuvant and adjuvant	1 (5.6)
Chemotherapy after RT only	1 (5.6)
None	1 (5.6)
ypT	
ypT1	1 (5.6)
ypT2	1 (5.6)
ypT3a	5 (27.8)
ypT3b	4 (22.2)
ypT4a	7 (38.9)
ypN	
ypN0	3 (16.7)
ypN1	4 (22.2)
ypN2	10 (55.6)
ypN3	1 (5.6)
Surgical margins	
Negative	10 (55.6)
Positive	8 (44.4)
LVI	
Negative	7 (38.9)
Positive	11 (61.1)
Dose, Gy	
50.4	9 (50.0)
55.8	6 (33.3)
Incomplete treatment	3 (16.8)

**Abbreviations:** RT, radiation therapy; LVI, lymphovascular invasion.

Mean baseline and postradiation CBC values are listed in Table 2. Notably, on average, patients were anemic at baseline (mean Hgb level, 10.5 g/dL), whereas all other values were within reference ranges. After completion of radiation therapy, WBC, ANC, ALC, and platelet counts were all significantly decreased (*P* < .01), whereas Hgb levels remained stable. Evaluating the absolute hematologic nadir during RT, neutropenia (ANC < 1.5 × 10^9^/L) developed in 1/16 assessable patients, lymphocytopenia (ALC < 1 × 10^9^/L) developed in 14/15 assessable patients, and thrombocytopenia (platelets < 150 × 10^9^/L) developed in 4/16 assessable patients.

**Table 2.** Association of mean blood counts before and after radiation therapy (RT).

	**Before RT (Mean ± SD)**	**After RT (Mean ±SD)**	***P*** **value^a^**
WBC	7.21 ± 1.31	4.78 ± 1.11	**< .001**
ANC	4.35 ± 1.52	3.17 ± 1.14	**< .001**
ALC	1.83 ± 0.59	0.64 ± 0.25	**< .001**
Hgb	10.52 ± 1.20	10.56 ± 1.25	.91
Platelets	273.44 ± 94.37	219.38 ± 112.63	**.03**

**Abbreviations:**, WBC, white blood cell count; ANC, absolute neutrophil count; ALC, absolute lymphocyte count; Hgb, hemoglobin.

a Bolded values are considered significant.

Correlations between PBM dose levels and changes in CBC values are listed in Table 3. Increasing mean PBM dose was associated with lower nadirs in WBC (Pearson CC −0.593, *P* = .02) and ANC (Pearson CC −0.597, *P* = .02). Increasing PBM volume (percentage) receiving 30 Gy (Pearson CC −0.618, *P* = .01) and 40 Gy (Pearson CC −0.512, *P* = .04) was associated with lower WBC nadir, and increasing PBM volume (percentage) receiving 20 Gy (Pearson CC −0.569, *P* = .03) and 30 Gy (Pearson CC −0.598, *P* = .02) was associated with lower ANC nadir. Mean PBM dose was also weakly correlated with lower Hgb nadir (Pearson CC −0.506, *P* = .046).

**Table 3.** Dosimetric correlations with cell count nadirs.

**PBM variable**	**Pearson CC**	***P*** **value^a^**
WBC		
Mean dose	−0.593	**.015**
V5, %	−0.073	.789
V10, %	−0.194	.472
V20, %	−0.398	.127
V30, %	−0.618	**.011**
V40, %	−0.512	**.043**
ANC		
Mean dose	−0.597	**.024**
V5, %	−0.012	.966
V10, %	−0.311	.279
V20, %	−0.569	**.034**
V30, %	−0.598	**.024**
V40, %	−0.380	.180
ALC		
Mean dose	0.213	.465
V5, %	0.163	.577
V10, %	0.436	.119
V20, %	0.466	.093
V30, %	−0.001	.998
V40, %	−0.105	.720
Platelets		
Mean dose	−0.281	.292
V5, %	0.083	.761
V10, %	0.069	.801
V20, %	−0.113	.678
V30, %	−0.324	.221
V40, %	−0.349	.185
Hgb		
Mean dose	−0.506	**.046**
V5, %	−0.045	.868
V10, %	−0.264	.324
V20, %	−0.385	.141
V30, %	−0.410	.115
V40, %	−0.415	.110

**Abbreviations:** PBM, pelvic bone marrow; CC, correlation coefficient; WBC, white blood cell; VXX, volume receiving XX Gy; ANC, absolute neutrophil count; ALC, absolute lymphocyte count; Hgb, hemoglobin.

a Bolded values are considered significant.

**Figure 2.**
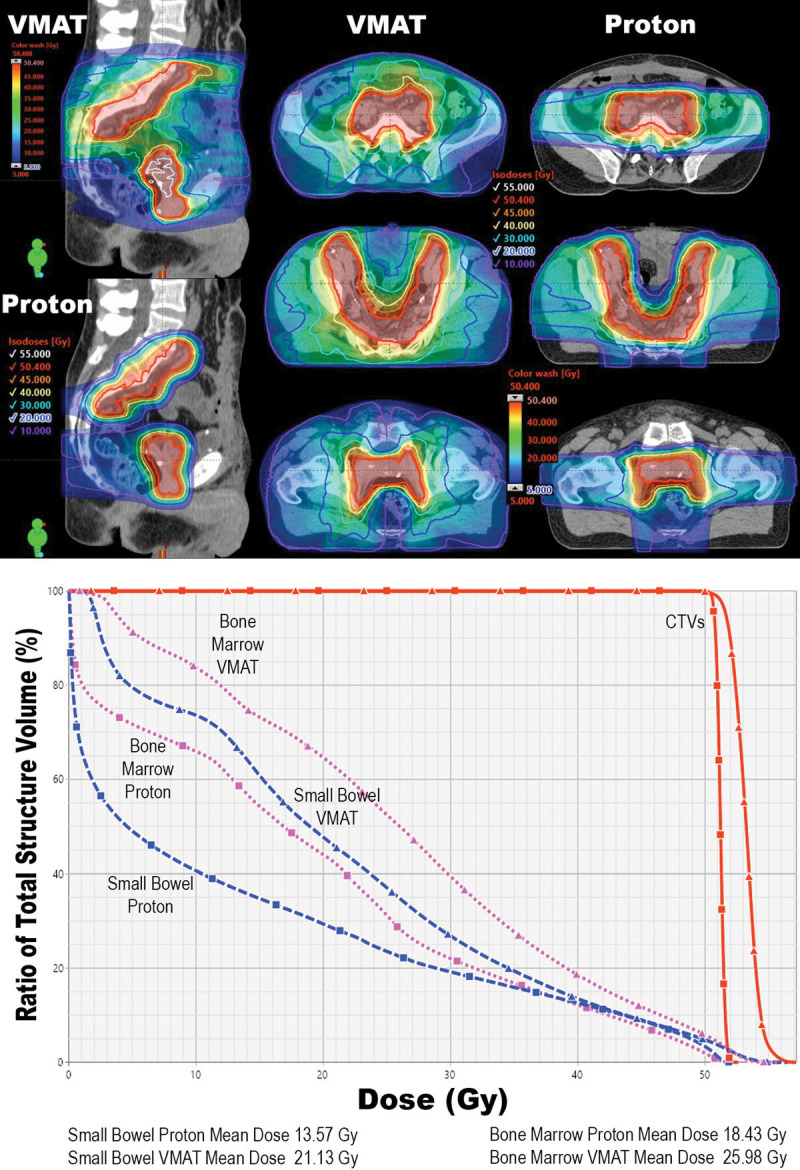
Sagittal and axial computed tomography slices of a study patient including radiation isodose lines and dose color wash of the delivered VMAT plan and a comparative PBT plan (top) and the representative DVH graphs (bottom). This patient with high-risk bladder cancer received postoperative radiation therapy to 50.4 Gy. Abbreviations: VMAT, volumetric moderated arc therapy; PBT, proton beam therapy; DVH, dose-volume histogram.

On dosimetric comparison of photon and PBT plans, PBT achieved significant reductions in mean, V5, V10, V20, V30, and V40 PBM dose (all *P* < .001) (**Figure 2**). Differences in photons and PBT plans included reduction in mean PBM dose from 26.5 Gy to 16.1 Gy and reduction in the mean PBM V20 from 63.9% to 34.1% (all *P* < .001). In addition, mean small-bowel dose (Gy) was significantly reduced from 21.7 Gy to 12.8 Gy (*P* < .001). Full details are listed in Table 4.

**Table 4.** Dosimetric comparison of proton beam therapy (PBT) and volumetric modulated arc therapy (VMAT) plans.

	**VMAT, mean ± SD**	**PBT, mean ± SD**	***P*** **value^a^**
Mean PBM dose, Gy	26.5 ± 1.8	16.1 ± 2.9	**< .001**
PBM V5, cm^3^	925.5 ± 195.9	692.9 ± 143	**< .001**
PBM V10, cm^3^	834.5 ± 181.3	595.33 ± 168.5	**< .001**
PBM V20, cm^3^	654.2 ± 127.0	352.8 ± 352.8	**< .001**
PBM V30, cm^3^	425.2 ± 82.2	194.3 ± 63.0	**< .001**
PBM V40, cm^3^	230.5 ± 65.1	103.77 ± 41.2	**< .001**
Mean small-bowel dose, Gy	21.7 ± 6.1	12.8 ± 5.0	**< .001**
Small-bowel V45, cm^3^	295.2 ± 175.6	234.2 ± 140.1	.26

**Abbreviations:** PBM, pelvic bone marrow; VXX, volume receiving XX Gy.

a Bolded values are considered significant.

## Discussion

Our secondary analysis of the ongoing prospective phase 2 clinical trial NCT01954173 (examining the role of adjuvant pelvic RT after radical cystectomy in bladder cancer using modern radiation techniques) [8], found an association between PBM dose and acute reductions in blood cell counts. These results corroborate reports from other pelvic malignancies that similarly correlate PBM dose with hematologic nadirs and clinical toxicity [9–11]. Understanding the clinical importance of excess dose to PBM, including mean and intermediate (V20-V40) dose metrics, presents an opportunity to further reduce treatment-related toxicity with PBT.

Locally advanced bladder cancer is a challenging disease, which often results in suboptimal outcomes. The concern for excessive treatment-related toxicities is, in large part, because this disease most commonly affects older patients with significant medical comorbidities. Adequate oncologic resection requires a radical cystectomy, which alone is associated with a 90-day complication rate of 30% and a 90-day mortality rate of 4% based on modern reports [12]. Adjuvant RT, despite clearly reducing LRF, has historically been discouraged because of elevated rates of late gastrointestinal toxicity, ranging from 36% to 59% [5, 13, 14]. However, these studies were primarily conducted in the 1980s using outdated 2-dimensional RT techniques and larger treatment fields, increasing unneeded dose to bowel.

Results from a recent Egyptian National Cancer Institute (NCI) trial [7] randomizing the use of 3-dimensional-conformal RT after radical cystectomy and chemotherapy showed promising 2-year results, including clear improvement in locoregional recurrence–free survival compared with adjuvant chemotherapy alone (96% versus 69%, *P* < .01) and trends towards improved disease-free survival (68% versus 56%, *P* = .07) and overall survival (71% versus 60%, *P* = .11). This trial also, importantly, reported a low rate of late grade 3^+^ toxicity of 7% [7]. Given these results, in addition to numerous other modern case series showing efficacy and tolerability of adjuvant pelvic RT [15–23], the National Comprehensive Cancer Network (NCCN; Plymouth Meeting, Pennsylvania) recently revised their guidelines to include the option to consider postoperative RT for patients with elevated risk of LRF based on pathologic risk factors, such as pT3 and pT4, node-positive, or positive-margin disease (category 2B) [24].

Another important reason adjuvant RT is underutilized is due to concerns for hematologic toxicity related to bone marrow suppression, which may delay and/or preclude administration of additional adjuvant systemic therapies. Bone marrow progenitor cells are highly radiation sensitive and have been associated as a primary cause of acute myelosuppression after 5 to 6 weeks of pelvic irradiation [9–11]. The destruction of circulating peripheral blood-pool stem cells also contributes to radiation-related cytopenia, but given the half-life of certain blood cells, such as leukocytes and platelets, is on the order of days, cell-count regeneration in the absence of bone marrow suppression would be expected within the study time frame [25, 26]. The Egyptian trial [7] reported moderate rates of acute hematologic toxicity, including 68% grade 2^+^ anemia, 13.3% grade 2^+^ neutropenia, and 4% grade 2^+^ thrombocytopenia in the RT arm. In the current study, by the end of radiation therapy, nearly all patients developed lymphocytopenia, and one fourth of patients developed thrombocytopenia. Although mixed dosimetric differences in bowel and rectal dose have been reported when comparing an older generation of PBT (single-field uniform dose-scanning technique) to intensity-modulated RT [27, 28], our study demonstrates a clear dosimetric advantage of pencil-beam scanning PBT in terms of dose sparing of the PBM. This is particularly true for low-to-moderate dose levels, which are most strongly associated with treatment-related cytopenia in other pelvic disease sites [9–11, 29, 30]. Minimizing hematologic toxicities can be clinically relevant not only to reduce acute clinical toxicity but also to increase adherence to systemic therapy regimens. This is especially true for an older and frail patient population that requires the use of multiagent chemotherapy. For example, 13% of patients on the RT arm of the Egyptian NCI trial [7] were unable to complete the entire prescribed therapy course despite the average age of enrolled patients in the trial being 54 years (and patients older than 70 years being excluded) [31]. In contrast, the average age of patients at diagnosis of bladder cancer is 73 years [32]. This discrepancy suggests application of PBT may be more useful in an older patient population that may be less able to tolerate aggressive adjuvant therapies.

This study has several limitations. Although having the advantage of evaluating patient data prospectively collected during a clinical trial, this study included a relatively small patient cohort and was unable to report physician- or patient-reported clinical toxicity to corroborate the clinical implications of these cell nadirs. These results will be reported in future primary analysis of NCT01954173 [8]. In addition, there was variability in sequencing and use of chemotherapy in this trial, potentially affecting cell-count dynamics. This study also used a geometric inner margin on the pelvic bony anatomy to define the PBM contour. Use of functional imaging, such as ^18^F-FDG (2-deoxy-2-[fluorine-18]fluoro-d-glugose) positron emission tomography to define active PBM may provide a more accurate avoidance target [33]. Ultimately, randomized trials will be required to confirm the clinical value of the dosimetric advantages seen with PBT planning. Although the cooperative group trial NRG GU001 (NCT02316548) [34] closed early because of poor accrual, other international randomized trials, including GETUG-AFU 30/Bladder-ART from France (NCT03333356) [35] and the Bladder Cancer Adjuvant Radiotherapy trial from India (NCT02951325) [36] are ongoing. We expect these to clarify the modern role of postoperative RT for bladder cancer and its associated risks of acute and late toxicities.

## Conclusion

Secondary analysis of the ongoing phase 2 clinical trial NCT01954173 [8] demonstrated increased dose (mean and V20-V40) to the PBM was associated with lower hematologic nadirs. Dosimetric comparisons between pencil-beam scanning PBT plans and the delivered VMAT plans produced > 40% reduction in mean PBM dose and a 46% reduction in the PBM V20. Therefore, PBT may be a valuable strategy to reduce the risk of hematologic toxicity and improve treatment adherence in this typically older and frail patient population. Further investigation to confirm the clinical significance of these findings is warranted.
